# In-office bleaching effect with different concentrations of hydrogen peroxide on enamel color and demineralization

**DOI:** 10.6026/973206300222395

**Published:** 2026-04-30

**Authors:** Bilal Ahmad Mir, Muneesa Jan, Anu Sharma, Aamir Rashid Purra, Fayaz Ahmed Ahanger, Ajaz Amin Masoodi

**Affiliations:** 1 Department of Conservative Dentistry and Endodontics, Government Dental College and Hospital, Srinagar, Jammu and Kashmir, India

**Keywords:** Colorimetry, DIAGNOdent, enamel demineralization, hydrogen peroxide(HP), split-tooth model, *in vitro* study, tooth bleaching

## Abstract

Use of hydrogen peroxide in tooth bleaching can cause microscopic alterations such as increasedsurface depressions, irregularities and elevated surface roughness. Therefore, it is of interest to report the enamel surface effects of two in-office hydrogen peroxide (HP) bleaching gels. Forty-eight intact human premolars were sectioned mesio-distally, creating two halves, which were then assigned to 2 groups. Group I (35% HP with calcium; whiteness HP Blue 35%, FGM) or Group II (40% HP with fluoride/potassium nitrate; Opalescence Boost, Ultradent). Bleaching was performed in a single 40-minute session per manufacturer protocol. Baseline and post-treatment measurements were taken using a spectrophotometer (VITA Easyshade) for color and a laser fluorescence device (DIAGNOdent pen, KaVo) for quantifying demineralization. The 40% hydrogen peroxide gel with fluoride and potassium nitrate produced a superior whitening effect; both bleaching agents resulted in comparable levels of enamel surface demineralization.

## Background:

In recent years, growing patient interest in appearance has led to increased demand for aesthetic dental treatments. Tooth bleaching represents a significant component of aesthetic dentistry and is regarded as the most effective, safe and conservative approach for managing tooth discoloration [1]. Commonly used bleaching agents include hydrogen peroxide (HP) and carbamide peroxide (CP). In-office procedures typically employ high-concentration HP (20-45%), low-concentration HP (<20%), or high- concentration CP (≥37%). When applied to the tooth, these oxidizing agents diffuse rapidly into enamel and dentin, decomposing into unstable free radicals. The bleaching effect occurs as these radicals degrade organic pigments, reducing their light reflection [2]. However, due to its low molecular weight, HP can penetrate tooth structure, reach the pulp chamberand induce inflammatory mediator release, resulting in tooth sensitivity [3, 4]. Additionally, HP can cause microscopic alterations such as increased porosity, surface depressions, irregularities, elevated surface roughness and reduced surface hardness [5, 6-7]. These undesirable outcomes depend on factors including bleaching gel composition, peroxide concentration, pH, application protocol and treatment duration [8]. To mitigate adverse effects associated with high-concentration HP, strategies such as incorporating remineralizing agents and reducing peroxide concentrations have been explored [9, 10]. Studies indicate that adding remineralizing compounds to high-concentration HP office bleaching gels can enhance enamel microhardness and reduce enamel erosion [11]. Recent research also supports the efficacy of low-concentration HP gels for bleaching, with *in vitro* studies reporting reduced cell damage and fewer surface changes at lower peroxide levels [12, 13]. In response, manufacturers have developed various bleaching agent concentrations and application methods to optimize outcomes, leading to a proliferation of commercial in-office bleaching products containing high levels of hydrogen peroxide (15-45%) or carbamide peroxide (≥37%). Since peroxide concentration and application technique are key determinants of bleaching effectiveness, research has aimed to harness saliva's natural remineralizing ability and, more directly, to include remineralizing additives in bleaching formulations. Compounds such as fluoride, calcium, phosphate and potassium nitrate have been suggested to help preserve enamel integrity by promoting mineral re-deposition and decreasing postoperative sensitivity [14, 15]. Therefore, it is of interest to evaluate and compare two commercially available in-office bleaching gels with differing HP concentrations and remineralizing additives 35% HP with calcium versus 40% HP with fluoride and potassium nitrate focusing on two outcomes: (1) effectiveness in enamel color change and (2) extent of enamel surface demineralization. A split-tooth design was utilized to improve comparison validity by minimizing inter-tooth variability.

## Null hypothesis:

Before start of the study null hypothesis was stated that, there was no significant difference in whitening efficacy or enamel demineralization between 35% HP with calcium and 40% HP with fluoride/potassium nitrate bleaching gels.

## Materials and Methods:

## Sample selection and preparation:

A total of 48 sound human premolars, freshly extracted for orthodontic or periodontal reasons, were collected under an ethically approved protocol. The inclusion criteria mandated teeth free from caries, cracks, hypoplastic defects and any form of restoration or staining (e.g., tobacco). Following extraction, teeth were cleaned with pumice slurry, stored in 0.1% thymol solution at 4°C and used within three months. Each tooth was sectioned longitudinally in a mesio-distal direction using a water-cooled diamond saw (IsoMet, Buehler) to create two symmetrical halves. This split-tooth model ensured that both test groups contained enamel from the same tooth origin, thereby minimizing the influence of inherent variations in mineralization, shade and morphology.

## Group allocation and bleaching procedure:

The 96 tooth halves were randomly assigned to one of two experimental groups (n=48 halves per group) using a computer-generated randomization list:

[1] Group I: Treated with 35% hydrogen peroxide gel containing calcium (Whiteness HP Blue 35%, FGM Dental Products, Joinville, SC, Brazil).

[2] Group II: Treated with 40% hydrogen peroxide gel containing 0.11% fluoride and 3% potassium nitrate (Opalescence Boost, Ultradent Products Inc., South Jordan, UT, USA). The bleaching procedure strictly followed the manufacturers' instructions for a single in-office session. The gel was applied in a 1-2 mm thick layer on the buccal enamel surface. For Group I, the gel was left for 40 minutes. For Group II, the gel was applied and reactivated every 20 minutes for a total of 40 minutes. After treatment, the gel was thoroughly removed with cotton pellets and water spray.

## Outcome measurements:

## Color assessment:

Baseline (T0) and post-bleaching (T1) color measurements were performed using a calibrated digital spectrophotometer (VITA Easyshade V, VITA Zahnfabrik). The device was positioned perpendicular to the central buccal surface. Color was recorded in the CIE L*a*b* system. The overall color difference (ΔE) was calculated using the formula: ΔE = √ [(ΔL*) ^2 + (Δa*) ^2 + (Δb*) ^2].

## Demineralization assessment:

Enamel surface mineralization status was evaluated using a laser fluorescence device (DIAGNOdent pen 2190, KaVo). The device was calibrated against a ceramic standard and then applied to the center of the bleached enamel surface. DIAGNOdent readings (scale 0-99) were recorded at T0 and T1. An increase in the numerical value indicates a decrease in relative mineral content (higher fluorescence due to porphyrins in demineralized areas).

## Statistical analysis:

Data were tabulated in Microsoft Excel and analyzed using SPSS Statistics version 20.0 (IBM Corp.). Normality of data distribution was confirmed using the Shapiro-Wilk test. Descriptive statistics were expressed as mean ± standard deviation (SD). Intergroup comparisons of ΔE values and changes in DIAGNOdent readings (ΔDD) were performed using the independent samples t-test. A p-value of less than 0.05 was considered statistically significant.

## Results:

Both groups exhibited a clinically perceptible color change (ΔE > 3.7). The whitening efficacy of Group II (40% HP) (6.65) was significantly greater than that of Group I (35% HP) (4.91). This difference was statistically highly significant (p < 0.001) (Table 1 and Figure 1 - see PDF). The DIAGNO dent readings increased from baseline in both groups, indicating a degree of surface demineralization post-bleaching. The mean change in DIAGNO dent value (ΔDD) was (12.7) for Group I and (13.4) for Group II. Statistical analysis revealed no significant difference in the degree of demineralization between the two groups (p = 0.187) (Table 2 and Figure 2 - see PDF). Parameter Group I (35% HP + CA) Group II (40% HP + F/KNO3) p-value.

## Discussion:

Hydrogen peroxide (HP) based bleaching agents remain the most preferred and effective treatment for tooth discoloration, primarily due to their potent oxidation reaction which degrades organic, chromogenic molecules within the enamel and dentin [16, 17 and 18]. However, this therapeutic efficacy is accompanied by well-documented alterations to dental hard tissues. A significant concern associated with bleaching is the potential for mineral loss. Research by Rotstein *et al.* (1999) demonstrated that both 10% carbamide peroxide and 30% hydrogen peroxide led to a significant reduction in the calcium-to-phosphorous ratio of dentin, indicating mineral dissolution [18]. This demineralization effect is not static but is directly influenced by the concentration of the bleaching agent and the duration of its application, as highlighted by Bistey *et al.* (2007) [19]. In alignment with manufacturer guidelines and to standardize the potential demineralization effect, the bleaching agents in the present study were applied for a controlled period of 40 minutes. To quantify this mineral change, our study utilized the DIAGNOdent device, a laser fluorescence tool validated for assessing the mineralization status of dental hard tissues [20]. The device provides a quantitative scale (0-99), where lower values correlate with higher mineral content and healthier tooth structure. While various methods exist to evaluate demineralization including visual inspection, photographic examination and fluorescent dye techniques, laser fluorescence offers an objective and non-invasive measurement [21]. Contrary to expectations based on the concentration-dependent relationship, our results indicated no statistically significant difference in mineral loss between the two experimental groups (35% HP and 40% HP). This suggests that within this high-concentration range and standardized application time, the demineralization effect may plateau or that the sensitivity of the device was insufficient to detect minor inter-group variations under the present experimental conditions. Concurrently, the efficacy of bleaching was assessed through shade evaluation. Spectrophotometers are recognized as highly accurate and practical tools for this purpose, offering advantages over digital cameras and colorimeters in objectivity and reliability [22, 23]. Our findings regarding efficacy revealed a notable outcome: Group 2 (40% HP) demonstrated a higher degree of discoloration compared to Group 1 (35% HP). This is consistent with the fundamental principle that higher peroxide concentrations generally yield more potent oxidizing effects. This result aligns with recent work by Altinisik *et al.* (2023), who also observed a concentration-dependent increase in bleaching efficacy [24]. Research has shown that even within the same mouth, teeth can have different compositions based on their function, exposure to environmental factors and individual biological variations. Differences in enamel thickness and dentin density among various tooth types within the same individual have been documented [25]. Biases like inter-tooth variability bias, location bias, selection bias or time lag bias can be encountered while comparing different remineralising agents. So in order to reduce bias as much as possible we have designed our own new innovative split tooth technique where each tooth was divided into two equal segments mesiodistally. Treatment was randomly assigned to either buccal or lingual part, enabling a direct comparison within the same tooth. This technique will remove a lot of inter-individual and inter-tooth variability as the comparison will be made on same tooth. Also fewer subjects will be required than parallel group comparison. The juxtaposition of our two primary findings presents a clinically relevant insight. While the higher concentration agent (40% HP) proved more effective in removing discoloration, it did not cause a statistically greater degree of demineralization than the 35% HP formulation under the tested parameters. This implies that, within the scope of this study, the increased efficacy may not come at the cost of disproportionately greater mineral loss. However, it is crucial to interpret this within the context of a single application period; repeated applications, as seen in clinical practice, could amplify demineralization effects over time. Future research should investigate the cumulative effects of multiple bleaching sessions and explore the potential of remineralizing agents, such as fluoride or calcium additives, to mitigate any mineral loss associated with these effective but chemically active treatments.

## Conclusion:

We show the 40% hydrogen peroxide gel with fluoride and potassium nitrate demonstrated significantly greater whitening efficacy than the 35% hydrogen peroxide gel. Both gels resulted in a comparable degree of enamel surface demineralization under the tested conditions. It is suggesting that the incorporated remineralizing agents may help buffer the potential adverse effects of higher peroxide concentrations. Therefore, for in-office bleaching, clinicians may consider gels with higher HP concentrations that are fortified with remineralizing agents.
